# Ocular tuberculosis in a calf.

**DOI:** 10.1186/s12917-021-02893-y

**Published:** 2021-05-08

**Authors:** Jacobo Carrisoza-Urbina, Mario A. Bedolla-Alva, Mireya Juárez-Ramírez, José A. Gutiérrez-Pabello

**Affiliations:** 1 Laboratorio de Investigación en Tuberculosis Bovina, Departamento de Microbiología e Inmunología, Facultad de Medicina Veterinaria y Zootecnia, Universidad Nacional Autónoma de México, Mexico city, Mexico; 2 Departamento de Patología, Facultad de Medicina Veterinaria y Zootecnia, Universidad Nacional Autónoma de México, Mexico city, Mexico

**Keywords:** Bovine tuberculosis, Meningeal tuberculosis, Ocular tuberculosis, Ocular granuloma

## Abstract

**Background:**

Bovine tuberculosis is a chronic inflammatory disease that causes granuloma formation mainly in retropharyngeal, tracheobronchial, mediastinal lymph nodes and lungs of bovines. The presence of these lesions in other tissues such as the eyeball is very rare and difficult to diagnose. This study describes macroscopic and microscopic pathological findings in a calf with ocular and meningeal tuberculosis.

**Case presentation:**

March 2019, an eight-month-old Holstein Friesian calf was identified in a dairy farm located in central Mexico with a clinical cough, anorexia, incoordination, corneal opacity and vision loss. At necropsy, pneumonia, lymphadenitis, meningitis, and granulomatous iridocyclitis were observed. The histopathological examination revealed granulomatous lesions in lung tissue, lymph nodes, meninges and eyes with the presence of acid-fast bacilli associated with *Mycobacterium spp.*

**Conclusion:**

To the best of our knowledge, this is the first report that describes macroscopic and microscopic pathological findings of ocular tuberculosis in cattle. This report highlights the importance of considering bovine tuberculosis in the differential diagnosis of corneal opacity and loss of vision in cattle.

## Background

Presence of extrapulmonary tuberculosis in humans in industrialized countries has increased from 16% in 1993 to 21% in 2006 [1]. The most lethal of these presentations is meningeal tuberculosis with an estimate of more than 100,000 cases per year [2]. Central nervous system involvement is a factor for the development of ocular tuberculosis, a very rare presentation in the population [3]. Likewise, cases of ocular tuberculosis in natural infections in animals are limited. Among the species susceptible to tuberculosis are cattle affected by *Mycobacterium bovis* (*M. bovis*) with a high prevalence in different parts of the world, showing similarities in immunopathology with tuberculosis in humans, which highlights the importance of this species as a model for studying ocular presentation [4–8]. Bovine tuberculosis frequently causes granulomatous lesions in the retropharyngeal, tracheobronchial, mediastinal lymph nodes, and lungs of cattle. Other less frequently affected organs are the regional lymph nodes, spleen, liver, kidney, intestine, mesenteric lymph nodes, vertebrae, and spinal cord. However, ocular involvement has rarely been reported [6, 8]. In this case report we present for the first time the macroscopic and microscopic pathological description of ocular tuberculosis in a calf.

## Case presentation

An eight-month-old Holstein Friesian calf from a stable with 220 cattle located in a complex of approximately 28,000 dairy cattle, with an intensive production system in the central area of Mexico, presented cough, anorexia, incoordination and loss of vision. The calf was referred to the Centro de Enseñanza y Diagnóstico en Enfermedades de los Bovinos of the Facultad de Medicina Veterinaria y Zootecnia at Universidad Nacional Autónoma de México, where the post mortem examination was performed. Bovines from this stable had previously been identified with lesions compatible with bovine tuberculosis.

**Fig. 1:**
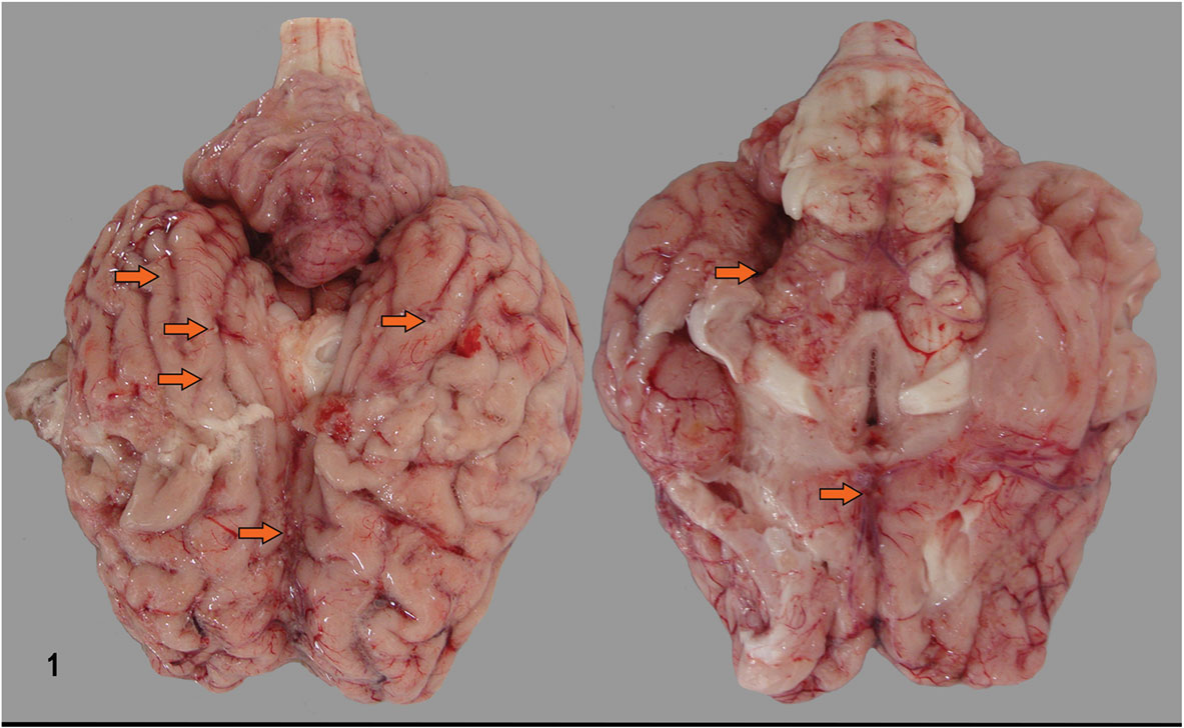
Meningeal tuberculosis in a bovine. Bovine brain with multifocal nodular granulomas in the meninges indicated by arrows

**Fig. 2:**
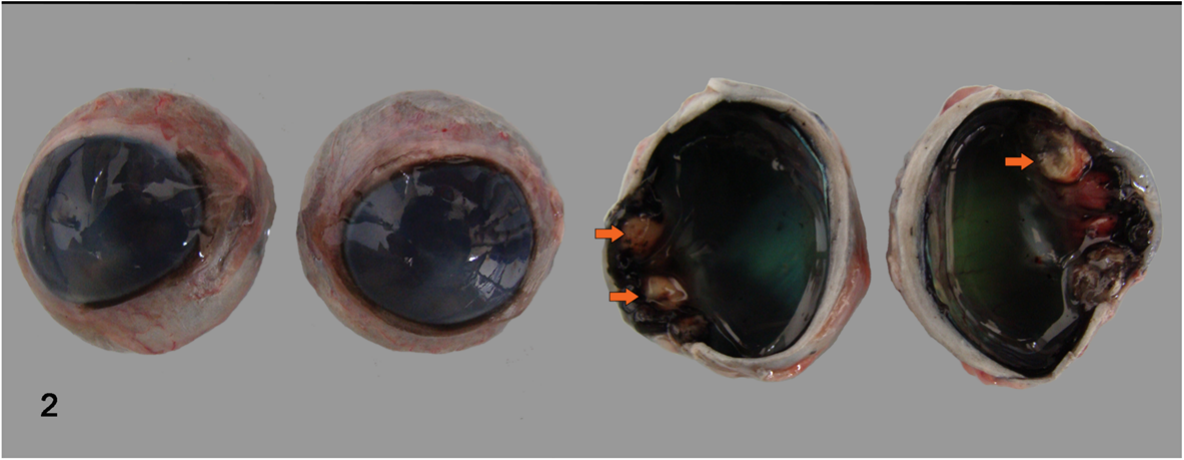
Ocular tuberculosis in a bovine. Corneal opacity in both eyes. Cross section of the eye shows anterior uvea thickening with nodular yellow coalescing granulomas indicated by arrows

Body inspection at necropsy revealed a carcass with pale conjunctival and oral mucosa. On internal inspection, the cranial, intermediate, accessory, and cranio-ventral lobes of the lungs were hyperemic and consolidated. The mediastinal lymph nodes were enlarged and extensive areas of yellow foci with granulomatous inflammation and a central core of caseous necrosis were identified. The leptomeninges presented many white nodules corresponding to granulomas in the cerebral hemispheres located mainly at the base of the brain (Fig. 1). Corneal opacity was observed in both eyes, the ciliary processes show thickening with nodular coalescing granulomas (Fig. 2).

**Fig. 3:**
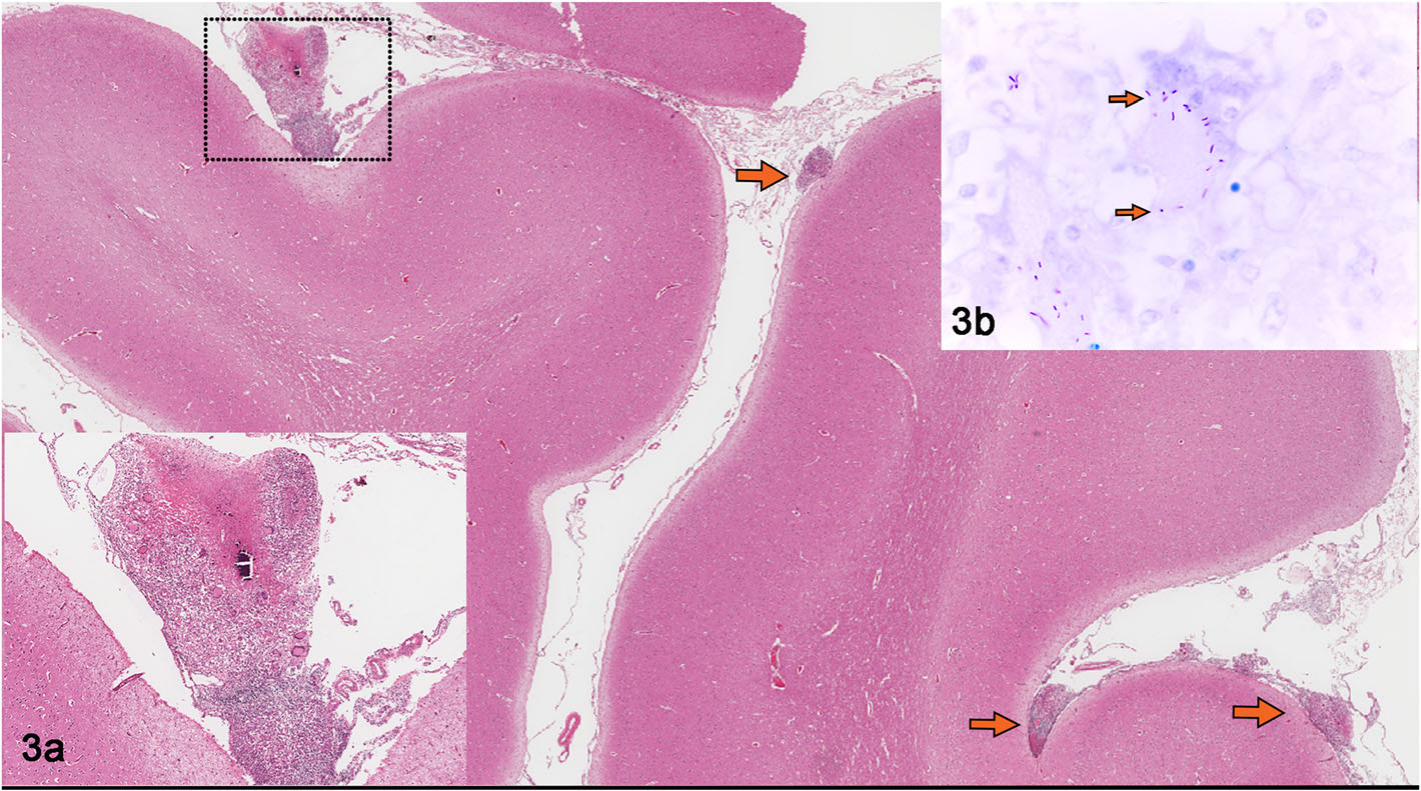
**a** Granulomas multifocal to coalescing in meningeal tuberculosis cause by *Mycobacterium spp*. in a bovine. **a** Section of the brain shows a granulomatous inflammatory infiltrate in the meninges indicated by arrows. The close-up shows a granuloma composed of numerous epithelioid macrophages and multinucleated giant cells, a necrotic area with mineralization and a thin fibrous connective tissue capsule. **b** Ziehl Neelsen stain shows acid fast bacilli indicated by arrows

**Fig. 4:**
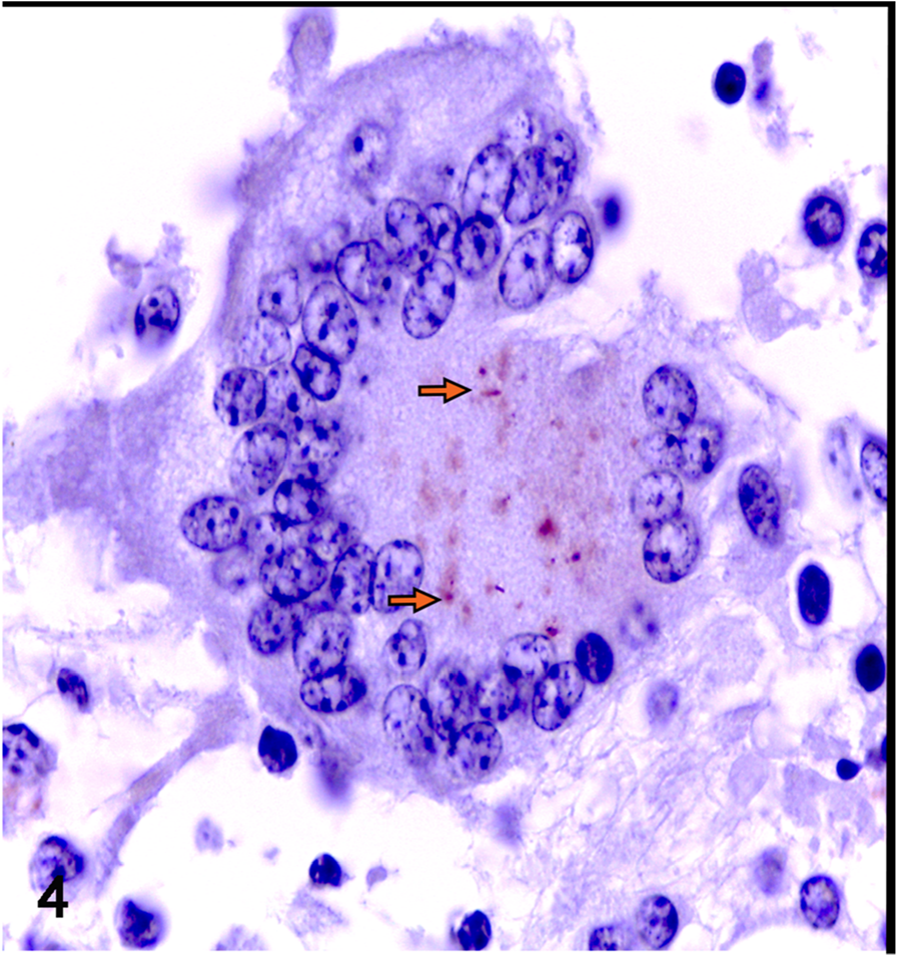
Immunohistochemistry of *Mycobacterium tuberculosis*. Shows a giant cell with cytoplasmic bacilli indicated by arrows in a section of the meninges

**Fig. 5:**
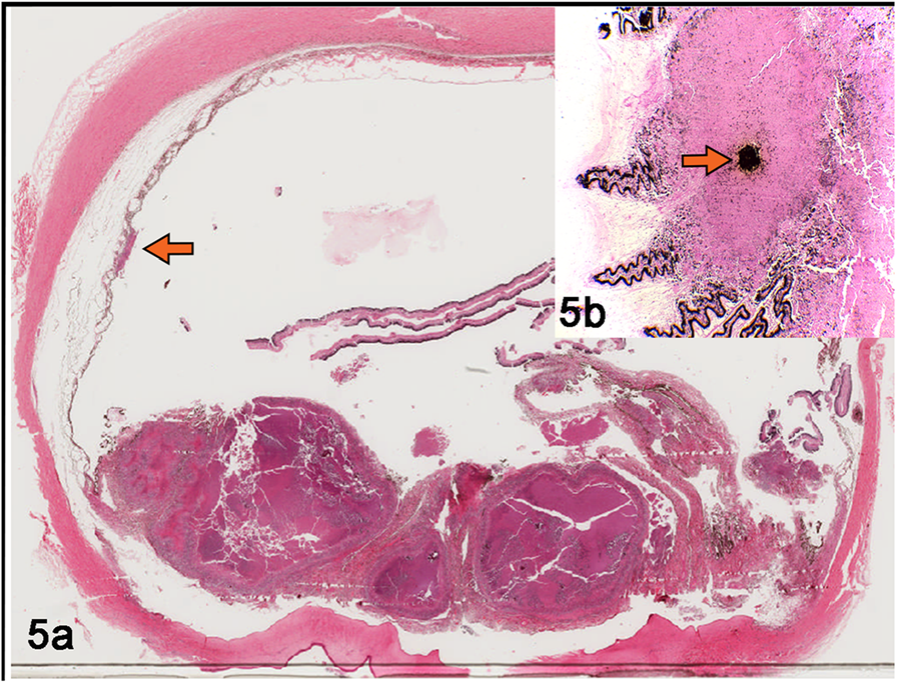
**a** Granulomas multifocal to coalescing in ocular tuberculosis cause by *Mycobacterium spp*. in a bovine. **a** Iris and ciliary body show coalescent granulomas with a thick capsule of connective tissue. The choroid shows mild granulomatous inflammation indicated by arrows. **b** Von Kossa stain shows mineralization in a granuloma indicated by arrows

Tissue segments from brain, eye, lymph node, and lung were fixed in 10% formaldehyde and paraffin-embedded for routine histological staining. A granulomatous inflammatory infiltrate was observed in the leptomeninges, mainly composed of macrophages, epithelioid macrophages, and lymphocytes. In some areas, granulomas were identified with necrotic center, surrounded by epithelioid macrophages, abundant multinucleated giant cells, interspersed with lymphocytes, plasma cells and few fibroblasts; some lesions contain central mineralization surrounded by numerous epithelioid macrophages (Fig. 3a). Bacilli were identified in areas with necrosis and in the cytoplasm of macrophages by Ziehl Neelsen (ZN) staining and immunohistochemistry (Figs. 3b and 4). The eyeball showed normal anatomic loss at the level of the lens, which was replaced by granulomatous lesions with similar characteristics as the lesions found in the meninges, showing rare neutrophils and a greater amount of connective tissue around the lesions. The choroid showed multifocal granulomas with acid fast bacilli in the macrophage cytoplasm and central mineralization identified by Von Kossa stain (Fig. 5a and b). In the mediastinal lymph nodes and lungs, granulomatous lesions with the characteristics previously described were also identified.

PCR using template DNA extracted from formalin-fixed paraffin-embedded (FFPE) tissues that had histopathological lesions compatible with tuberculosis was performed [9]. We used a universal set of primers spanning for V1-V3 short variable regions of the bacterial 16S rRNA gene that amplify a product of ~ 500 bp. A nested PCR for mpb70/m22 genes was used to identify members of the *Mycobacterium tuberculosis* complex, a positive result produces amplicons of 372 bp and 208 bp of length, respectively. In addition, an endpoint PCR of the RD9 with a product of 333 bp for *Mycobacterium tuberculosis* (*M. tuberculosis*) and 206 bp for *M. bovis* was performed*.* Finally, the RD4 gene was used to amplify a product of 268 bp for *M. bovis* and 172 bp for the rest of the members of the *Mycobacterium tuberculosis* complex*.* All primer sequences used are described in Table 1. Electrophoresis in a 2% agarose gel with SYBR Green (S9430 SIGMA-ALDRICH) was used for separated the products of DNA and next were visualized using a photo documenter (Gel Logic 200 Imaging System, Kodak, UK). Unfortunately, despite carrying out the different PCR protocols, it was not possible to obtain amplifications from the tissues of this case.

**Table 1:** The primers and sequencing used for PCR

Primers	Sequence (5′ → 3′)	Reference
16S-F	TTGGAGAGTTTGATCMTGGCTC	[10]
16S-R	GTATTACCGCGGCTGCTG	
MPB70-F	GAACAATCCGGAGTTGACAA	[11]
MPB70-R	AGCACGCTGTCAATCATGTA	
M22 -F	GAACAATCCGGAGTTGACAA	[11]
M22-R	CGTTGGCCGGGCTGGTTTGGCC	
RD9- F	GTGTAGGTCAGCCCCATCC	[12]
RD9-I	CAATGTTTGTTGCGCTGC	
RD9 -R	GCTACCCTCGACCAAGTGTT	
RD4-F	ATGTGCGAGCTGAGCGATG	[13]
RD4-I	TGTACTATGCTGACCCATGCG	
RD4-R	AAAGGAGCACCATCGTCCAC	

## Discussion and conclusions

According to the macroscopic findings at necropsy and laboratory tests performed, this case report describes a calf with bovine tuberculosis. The main finding was the presence of mycobacterial granulomatous lesions in the brain and both eyes. Although we attempted to identify the mycobacterium species by isolation or by polymerase chain reaction (PCR), all our efforts were negative. Therefore, we decided to use a polyclonal antibody against *M. tuberculosis* (biocare medical, CP 140) which cross reacts with members of the tuberculosis complex. Immunohistochemistry revealed a positive staining of mycobacterial antigens in the granulomatous lesions. Furthermore, *M. bovis* has previously been isolated in the calf herd and prevalence of bovine tuberculosis was greater than 16%, altogether our results suggests *M. bovis* as the etiological agent [14, 15]. The pathogenesis of ocular tuberculosis is unknown, until now it is believed that the spread of the bacteria originates from the primary site of infection to the eyeball by hematogenous route, frequently presenting granulomas in the choroid [8]. Endogenous infection of the eyeball by *M. bovis* has been identified in adults or immunocompetent people [16, 17]. Likewise, this affectation was reported in 3.2% (9/282) of patients who presented disseminated tuberculosis when receiving immunotherapy with Bacillus Calmette-Guérin (BCG) as a treatment for bladder cancer, causing uveitis, endophthalmitis and autoimmune retinopathy. This presentation differs from destructive intraocular tuberculosis caused by *M. tuberculosis*, which mainly originates choroidal granulomas and subretinal abscesses [18, 19].

Study of ocular tuberculosis in naturally infected animals will provide a better understanding of bovine tuberculosis pathogenesis. This report highlights the importance of considering bovine tuberculosis in the differential diagnosis of corneal opacity and loss of vision in cattle.

## Data Availability

The datasets used and/or analysed during the current study are available from the corresponding author on reasonable request.

## Abbreviations

FFPE: Formalin-fixed paraffin-embedded
HE: Hematoxylin and eosin staining method
PCR: Polymerase chain reaction
ZN: Ziehl-Neelsen staining method
